# Supplementary material for: Engineered transcription-associated Cas9 targeting in eukaryotic cells
Source: Nat Commun. 2024 Nov 27;15:10287. doi: 10.1038/s41467-024-54629-9 (PMC11603292; doi:10.1038/s41467-024-54629-9)
Supplement: Supplementary file 2 — Description of Additional Supplementary Files [file 41467_2024_54629_MOESM2_ESM.pdf]

Description of Additional Supplementary Files

File Name: Supplementary Data 1
Description: MNase-seq analysis of β-E-induced changes at individual nucleosomes within the *LYS2* ORF and across chromosome II

File Name: Supplementary Data 2
Description: List of *S. cerevisiae yeast* strains used in this work

File Name: Supplementary Data 3
Description: Circular plasmid DNA used in this work

File Name: Supplementary Data 4
Description: Sequences of commercially synthesized linear single-stranded DNA oligonucleotides used in experimental assays and human cell culture work

File Name: Supplementary Data 5
Description: Linear double-stranded DNA fragments used for CRISPR-assisted strain construction or donor-dependent editing assays in yeast

File Name: Supplementary Data 6
Description: Raw results from analyses of human cell culture deep sequencing data (output from IDT's CRISPR Analysis Tool).
