# Supplementary material for: Engineered transcription-associated Cas9 targeting in eukaryotic cells
Source: Nat Commun. 2024 Nov 27;15:10287. doi: 10.1038/s41467-024-54629-9 (PMC11603292; doi:10.1038/s41467-024-54629-9)
Supplement: Supplementary file 9 — Reporting Summary [file 41467_2024_54629_MOESM9_ESM.pdf]

nature portfolio

Corresponding author(s): Jef D. Boeke, Gregory W. Goldberg, Marcus B. Noyes

Last updated by author(s): Oct 9, 2024

Reporting Summary

Nature Portfolio wishes to improve the reproducibility of the work that we publish. This form provides structure for consistency and transparency in reporting. For further information on Nature Portfolio policies, see our Editorial Policies and the Editorial Policy Checklist.

Statistics

For all statistical analyses, confirm that the following items are present in the figure legend, table legend, main text, or Methods section.

n/a	Confirmed	
☐	☒	The exact sample size (*n*) for each experimental group/condition, given as a discrete number and unit of measurement
☐	☒	A statement on whether measurements were taken from distinct samples or whether the same sample was measured repeatedly
☐	☒	The statistical test(s) used AND whether they are one- or two-sided<br>*Only common tests should be described solely by name; describe more complex techniques in the Methods section.*
☒	☐	A description of all covariates tested
☐	☒	A description of any assumptions or corrections, such as tests of normality and adjustment for multiple comparisons
☐	☒	A full description of the statistical parameters including central tendency (e.g. means) or other basic estimates (e.g. regression coefficient) AND variation (e.g. standard deviation) or associated estimates of uncertainty (e.g. confidence intervals)
☐	☒	For null hypothesis testing, the test statistic (e.g. F, t, r) with confidence intervals, effect sizes, degrees of freedom and P value noted<br>*Give P values as exact values whenever suitable.*
☒	☐	For Bayesian analysis, information on the choice of priors and Markov chain Monte Carlo settings
☒	☐	For hierarchical and complex designs, identification of the appropriate level for tests and full reporting of outcomes
☒	☐	Estimates of effect sizes (e.g. Cohen's *d*, Pearson's *r*), indicating how they were calculated

Our web collection on statistics for biologists contains articles on many of the points above.

Software and code

Policy information about availability of computer code

Data collection	Raw next generation sequencing data was collected using Illumina NextSeq 2000, MiSeq, or NovaSeq X Plus instruments (commercial), raw flow cytometry data was collected using Sony SA3800 spectral cell analyzer or Sony SH800 cell sorter instruments (commercial), and raw attenuance measurements from yeast cultures grown in 96-well plates were collected using a BioTek Cytation 5 microplate reader (commercial).
Data analysis	Demultiplexed sequencing data from yeast allelic editing experiments or human cell culture indel experiments were analyzed using IDT's commercial rhAmpSeq CRISPR Analysis Tool (CRISPAltRations 1.1.0), which only required pre-processing of '.fastq' files when analyzing single-read NextSeq 2000 data; processing was performed with the Seqtk script for python. Demultiplexed sequencing data from MNase-seq experiments were processed and analyzed using open source code: Trimmomatic (v0.39) and FastQC (v0.11.4) for read processing and the Burrows Wheeler aligner mem algorithm (v0.7.7) and DANPOS (v2) pipeline for alignment/analyses. Demultiplexed sequencing data from ChIP-seq experiments were processed and analyzed using open source code: FastQC, Trim Galore (Babraham Bioinformatics), and Trimmomatic for read processing, bowtie2 and samtools for alignment/mapping, and bamCompare (deepTools) for analyses. Growth curve data and colony sizes were analyzed using custom python scripts and the following open source packages: SciPy, scikit-image, and seaborn. All quantitative plots presented in this work were generated using commercial or open source software: GraphPad Prism (commercial), Microsoft Excel (commercial), matplotlib for python (open source), DANPOS v2 (open source), or IGV (open source).

For manuscripts utilizing custom algorithms or software that are central to the research but not yet described in published literature, software must be made available to editors and reviewers. We strongly encourage code deposition in a community repository (e.g. GitHub). See the Nature Portfolio guidelines for submitting code & software for further information.

Data

Policy information about availability of data

All manuscripts must include a data availability statement. This statement should provide the following information, where applicable:

- Accession codes, unique identifiers, or web links for publicly available datasets
- A description of any restrictions on data availability
- For clinical datasets or third party data, please ensure that the statement adheres to our policy

Relevant data supporting the findings of this study are available in the published article, its supplementary files, and the Sequence Read Archive of the NCBI (BioProject accession codes: PRJNA1149169 and PRJNA1149094). Source data are provided with this paper.

Research involving human participants, their data, or biological material

Policy information about studies with human participants or human data. See also policy information about sex, gender (identity/presentation), and sexual orientation and race, ethnicity and racism.

Reporting on sex and gender	n/a (no research on human subjects was performed in this study)
Reporting on race, ethnicity, or other socially relevant groupings	n/a (no research on human subjects was performed in this study)
Population characteristics	n/a (no research on human subjects was performed in this study)
Recruitment	n/a (no research on human subjects was performed in this study)
Ethics oversight	n/a (no research on human subjects was performed in this study)

Note that full information on the approval of the study protocol must also be provided in the manuscript.

Field-specific reporting

Please select the one below that is the best fit for your research. If you are not sure, read the appropriate sections before making your selection.

☒ Life sciences ☐ Behavioural & social sciences ☐ Ecological, evolutionary & environmental sciences

For a reference copy of the document with all sections, see nature.com/documents/nr-reporting-summary-flat.pdf

Life sciences study design

All studies must disclose on these points even when the disclosure is negative.

Sample size	Statistical testing to predetermine experimental sample sizes was not performed in this work; sample sizes were chosen based on existing procedures and standards in the field.
Data exclusions	No samples were excluded from data analyses. Next generation sequencing analyses adhered to previously established read exclusion criteria based on read quality and/or mapping quality scores. For indel analyses performed on single-read NextSeq data, usage of IDT's rhAmpSeq CRISPR Analysis Tool (CRISPAltRations 1.1.0) required us to first sub-sample 0.100 million random reads for analysis; thus, the remaining reads were arbitrarily excluded from our analyses. Because this sub-sampling was performed randomly (using the Seqtk script for python) and was sufficiently large, similar results would be expected if all of the reads were considered for those analyses.
Replication	All assays were performed with at least three biological replicates except where noted otherwise. Biological replicates were performed on separate days for most assays except as noted for plate reader growth assays (yeast cells), mCherry/EGFP fluorescence FACS assays (yeast/human cells), and cell culture indel assays (human cells).
Randomization	Randomization was generally not relevant to this study because it did not involve human or animal subjects, and because most experiments were designed to test genetic variables on otherwise-isogenic backgrounds; all samples could be treated equivalently in such cases. In other cases where aliquot samples were withdrawn from a single starting population (clonal population or pool of transformants) for treatment under different downstream conditions, pipetting of equivalent volumes from a well-mixed liquid starting population effectively allowed for random sampling.
Blinding	Investigators were not blinded during data collection because the experiments did not involve human or animal subjects and were readily controlled without blinding. Investigators were not blinded during data analyses because the key findings are supported by quantitative measurements (with statistical testing where relevant) that do not rely heavily on subjective judgment for interpretation.

Reporting for specific materials, systems and methods

We require information from authors about some types of materials, experimental systems and methods used in many studies. Here, indicate whether each material, system or method listed is relevant to your study. If you are not sure if a list item applies to your research, read the appropriate section before selecting a response.

Materials & experimental systems

n/a	Involved in the study
☐	☒ Antibodies
☐	☒ Eukaryotic cell lines
☒	☐ Palaeontology and archaeology
☒	☐ Animals and other organisms
☒	☐ Clinical data
☒	☐ Dual use research of concern
☒	☐ Plants

Methods

n/a	Involved in the study
☐	☒ ChIP-seq
☐	☒ Flow cytometry
☒	☐ MRI-based neuroimaging

Antibodies

Antibodies used	Rabbit IgG Polyclonal anti-HA antibody from Abcam (ab9110)
Validation	Experimentally validated as 'ChIP Grade' by the manufacturer (Abcam) and cited in numerous studies for use with several species including S. cerevisiae

Eukaryotic cell lines

Policy information about cell lines and Sex and Gender in Research

Cell line source(s)	The U2OS.EGFP human (female) cell line was received from the Joung lab, Mass. General Hospital; all derivatives of U2OS.EGFP used in this work were generated via CRISPR-assisted editing followed by single-cell FACS to isolate clones.
Authentication	The parent U2OS cell line previously received STR profiling by ATCC and deep sequencing authentication; all derivatives generated in this work via CRISPR editing were genotyped by PCR and Sanger sequencing at the target locus.
Mycoplasma contamination	The U2OS.EGFP cell line was previously confirmed negative for Mycoplasma contamination and it was re-tested alongside its U2OS.EGFP::5' UTR-boxB2x derivative while culturing cells for EGFP fluorescence FACS assays; both were found negative. The CRISPR-edited U2OS.EGFP::Intron-boxB2x(V1.0) derivative generated in this study remains untested for Mycoplasma contamination.
Commonly misidentified lines (See ICLAC register)	n/a (No commonly misidentified cell lines were used in the study.)

Plants

Seed stocks	n/a (No research on plants was conducted in this study.)
Novel plant genotypes	n/a (No research on plants was conducted in this study.)
Authentication	n/a (No research on plants was conducted in this study.)

ChIP-seq

Data deposition

☐ Confirm that both raw and final processed data have been deposited in a public database such as GEO.

☐ Confirm that you have deposited or provided access to graph files (e.g. BED files) for the called peaks.

Data access links	Processed ChIP-seq coverage data is presented visually within the manuscript. Raw sequencing data files are deposited in the

Data access links<br>*May remain private before publication.*	Sequence Read Archive (SRA) of the NCBI (BioProject accession code: PRJNA1149094). https://www.ncbi.nlm.nih.gov/sra/?term=PRJNA1149094
Files in database submission	48 paired-end Illumina sequencing data files, representing all 24 samples used for ChIP-seq analyses in this work (12 immunoprecipitation samples and their corresponding 12 input samples).
Genome browser session<br>(e.g. UCSC)	n/a (UCSC genome browser not used)

Methodology

Replicates	All ChIP-seq coverage plots show data from single biological replicates (n = 1)
Sequencing depth	Between 39 and 70 million pairs of reads (2 x 51 bp) were obtained for each sample, of which at least 20 million pairs were uniquely mapped after filtering for quality. This corresponds to at least 170x mapping coverage, given the yeast genome size of ~12 Mb.
Antibodies	Rabbit IgG Polyclonal anti-HA antibody from Abcam (ab9110)
Peak calling parameters	n/a (Peak calling was not performed)
Data quality	All ChIP-seq data was obtained from single experiments and presented with minimal processing as described in the Methods. Data quality was ensured by analyzing the 'input' samples on an Agilent TapeStation 4150 prior to sequencing (to confirm DNA shearing down to fragment lengths within our expected range), and, by sequencing at a depth that allowed us to obtain at least 20 million pairs of uniquely mapped reads for every sample.
Software	Raw ChIP-seq data was collected using Illumina's commercial NovaSeq X Plus platform. Analysis was performed with various open-source software: bowtie2 to align reads, samtools to filter for reads with a minimum mapping quality of 30 and retain only properly paired reads, bamCompare from the package deepTools to normalize for read depth (Counts Per Million) and produce a final output as the ratio of IP coverage over input coverage in both bigwig and bedgraph formats, and matplotlib to generate the parent plots that were further compiled and annotated using commercial Adobe Illustrator software.

Flow Cytometry

Plots

Confirm that:

☒ The axis labels state the marker and fluorochrome used (e.g. CD4-FITC).

☒ The axis scales are clearly visible. Include numbers along axes only for bottom left plot of group (a 'group' is an analysis of identical markers).

☒ All plots are contour plots with outliers or pseudocolor plots.

☒ A numerical value for number of cells or percentage (with statistics) is provided.

Methodology

Sample preparation	Yeast samples were aliquoted for analysis from saturated cultures grown in synthetic complete (SC) media formulations, adherent human U2OS-derived cell lines were grown in DMEM-based media and detached from individual wells of a 6-well plate immediately prior to analyses; see relevant Methods sections for more details.
Instrument	Sony SA3800 spectral cell analyzer (Model #: LE-SA3800FA); Sony SH800 cell sorter (Model #: LE-SH800 SFP)
Software	Commercial software provided with the Sony SA3800 and SH800 instruments
Cell population abundance	30,000 gated events were analyzed from each sample of yeast cells and at least 19,000 gated events were analyzed from each sample of human cells; in each case, the gated events constitute a majority of the total events analyzed
Gating strategy	For analysis of yeast, only one gate was applied in order to exclude a minority of the total events that fell outside the expected range for our yeast cells (based on FSC-A & SSC-A measurements). For analysis of human cells, two gates were applied: the first excluded a minority of the total events that fell outside the expected range for our cells (based on FSC-A & BSC-A measurements), the second excluded doublet events (based on FSC-H & FSC-A measurements) to enrich for events containing single-cells.

☒ Tick this box to confirm that a figure exemplifying the gating strategy is provided in the Supplementary Information.
